# Immune surveillance for six vaccinable pathogens using paired plasma and dried blood spots in HIV infected and uninfected children in Kinshasa

**DOI:** 10.1038/s41598-022-12052-4

**Published:** 2022-05-13

**Authors:** A. Rodríguez-Galet, M. Rubio-Garrido, A. Valadés-Alcaraz, M. Rodríguez-Domínguez, J. C. Galán, A. Ndarabu, G. Reina, A. Holguín

**Affiliations:** 1 HIV Molecular Epidemiology Laboratory, Microbiology Department, Ramón y Cajal University Hospital-IRYCIS-CIBERESp-RITIP-CoRISpe, Carretera de Colmenar, Km.9, 100, 28034 Madrid, Spain; 2 Microbiology Department, Ramón y Cajal University Hospital, Madrid, Spain; 3 CIBER in Epidemiology and Public Health (CIBERESP), Madrid, Spain; 4 Monkole Hospital, Kinshasa, Democratic Republic of the Congo; 5 Microbiology Department, University of Navarra Clinic-IdiSNA, Pamplona, Spain

**Keywords:** HIV infections, Paediatric research, Immunology, Infectious diseases, Vaccines

## Abstract

Child vaccination reduces infant mortality rates. HIV-infected children present higher risk of diseases than non-infected. We report the protection coverage rates for 6 vaccine-preventable diseases in a paediatric population from the Democratic Republic of the Congo (DRC) and the impact of HIV infection, providing the first data on the validity of dried blood samples (DBS) to monitor the immune protection. During 2016–2018 DBS from 143 children/adolescents were collected in Kinshasa (DRC), being 52 HIV-infected. Forty-two had a paired plasma sample. Protective IgG was quantified (VirClia-IgG,VIRCELL) to obtain the optimal cut-off in IgG detection in DBS. ROC curves were generated with R software and statistical analyses with Stata. Protective IgG levels varied across pathogens, not reaching herd immunity. HIV-infected presented lower vaccine protection than uninfected for all analyzed pathogens, except rubella, with statistically significant differences for measles (30.8% vs. 53.8%; *p* = 0.008) and tetanus (3.8% vs. 22%; *p* = 0.0034). New cut-offs were calculated when using DBS to improve test performance. We reinforce the necessity to increase pediatric vaccination coverage in Kinshasa, especially in HIV seropositive, with less capacity to maintain adequate antibody levels. DBS were useful to monitor vaccination coverage in seroprevalence studies in resource-limited settings, after optimizing the cut-off value for each pathogen.

## Introduction

Child vaccination is the most cost-effective public health intervention available to reduce the morbi-mortality in children and adolescents^1^. In the last three decades vaccination coverage against vaccine-preventable infectious diseases has increased, reducing global deaths by almost 60% from 1990 to 2019; however, there are still 20 million children in the world who do not receive essential vaccines such as diphtheria-tetanus-pertussis-containing vaccine (DTP) or measles vaccine. The 39% of unvaccinated children live in five countries: Democratic Republic of the Congo (DRC), Nigeria, India, Pakistan and Ethiopia^1^, hosting 49% of children’s death under 5 years worldwide^2,3^.

The DRC presents high mortality rate in children under 5 years, reaching 85 per 1000 births^4^. According to the Multiple Indicator Cluster Survey, 65% of children under 2 years in the DRC had not received any vaccine or were incompletely vaccinated during 2018–2019^5,6^. The absence of correct immunity in the paediatric population causes periodic outbreaks of vaccine-preventable diseases in the country^7^.

The immunization is the process of inoculating a vaccine to protect against infection and/or disease^8^. During 2019, approximately 85% of infants around the world received 3 doses of the DTP vaccine, used as an indicator of the ability of countries to provide immunization services^1^. According to 2020 WHO data for the DRC, the coverage of DTP third doses (DTP3) and measles-containing-vaccine (MCV) was 57%, with no data of rubella vaccine coverage. The DRC vaccination schedule does not yet contemplate the second dose of MCV^9^, used to assess the ability to continue immunization services between the second and fifth year of life^3^.

Children living with the human immunodeficiency virus (HIV) tend to have lesser vaccine protection against vaccine-preventable diseases vs. unexposed children^10^. Thus, it is important to optimize the vaccination schedule, especially in countries with a high rate of paediatric HIV infections. Protective titers after vaccine immunization in HIV-infected children varies depending on the vaccine, immune recovery and viral suppression^11^. Revaccination after initiating antiretroviral therapy (ART) could help to increase protection in HIV-infected children^12,13^. Most of them live in low-middle income countries, where scarce laboratory resources and limited conditions for collecting and handling serum/plasma from blood, which makes difficult to monitor the real paediatric vaccination coverage. In these circumstances, dried blood samples (DBS) could represent a convenient sample for antibody detection^14^, being easy to collect, store and transport without cold chain^15^. DBS have been used in the surveillance and monitoring of numerous infectious diseases^14–20^. The good correlation between antibody concentrations in DBS and serum/plasma samples support the wider use of DBS in post-vaccination and seroepidemiological studies^14^.

Our study reports the immune seroprotection rates to 6 vaccine-preventable diseases (diphtheria, tetanus, pertussis, measles, mumps, rubella) in a paediatric and adolescent population in Kinshasa (DRC), analysing the impact of HIV infection. Furthermore, we provide the first data on the validity of DBS to test the immune protection to these 6 pathogens, establishing the cut-off values that provide optimal sensitivity and/or specificity in DBS for each infection in the study cohort.

## Methods

### Clinical and laboratory procedures

DBS from 143 children and adolescents under clinical follow-up in Monkole and Kalembelembe hospitals (Kinshasa, DRC) were collected between 2016 and 2018, as previously described^21^. Forty-two of them (29.4%) also had paired plasma. All specimens had associated clinical-epidemiological data. The samples were stored in Kinshasa hospitals at − 20 °C until their transport to Madrid, where they were kept at − 80 °C until processing to analyse and quantify the presence of protective IgG against six pathogens responsible for vaccine-preventable diseases: diphtheria, tetanus, pertussis, measles, mumps and rubella.

The first HIV diagnosis was performed in Kinshasa by three rapid serological tests: Determine™HIV-1/2Ag/Ab (ALERE), Double-Check Gold HIV1&2 (ORGENICS) and Uni-Gold HIV (TRINITY BIOTECH) in patients older than 18 months of age. In infants under 18 months, the 4th generation immunoassay VIDAS® HIV Duo Ultra (BIOMERIEUX) or, exceptionally, the molecular test ABBOTT real-time HIV-1 Qualitative were used.

In the HIV-1 Molecular Epidemiology Laboratory (Madrid), the serological status of the children was confirmed by the Geenius™HIV-1/2 (BIO-RAD) assay. One whole blood spot was taken from each card and eluted in 150 µl NucliSens easyMAG elution buffer 3 (BIOMERIEUX) for 1 h with rotation. The test was performed according to manufacturer indications, but using 40 µl of the eluted, as previously published by our group^19^. The results obtained by Geenius™HIV-1/2 were confirmed in all seropositive and indeterminate patients using the molecular point-of-care XpertQual (CEPHEID), which provides a binary “detected”/“not detected”, according to manufacturer’s instructions.

### Quantification of IgG against six vaccinable diseases

VirClia-IgG (VIRCELL) technique, an indirect chemiluminescent immunoassay to detect the antibodies present in the sample with the antigen bound to the polystyrene surface, was used to quantify the presence of protective IgG against the six pathogens with 5 µl of plasma. When using DBS, one dot (70 µl of whole blood containing 42.7 µl plasma after considering 39% haematocrit^22^) was eluted in 770 µl of phosphate-buffered saline (PBS), being tested 100 µl of the final elution in each specific VirClia-IgG test (equivalent to 5 µl plasma per test). We considered as gold standard the cut-off values provided by VirClia for the six pathogens in plasma/serum (Table 1), which provides 96–100% sensitivity and 100% specificity. We considered that a child had protective IgG levels for a pathogen under study, or concentration of antibody titers with sufficient affinity required to neutralize the pathogen^8^, when the anti-pathogen IgG levels in his plasma were included in the range of positivity proposed by VIRCELL and specified in the technical file of each specific VirClia-IgG assay.

**Table 1:** VirClia-IgG (VIRCELL) cut-off values for plasma/serum.

	Semi-quantitative (IU/ml)			Qualitative (index)		
	Pertussis	Diphtheria	Tetanus	Measles	Mumps	Rubella
Positive	> 120	≥ 0.1 Max. protection	> 0.2	1.1	1.1	1.1
		0.01–0.09 Basic protection				
Indeterminated	60–120	–	0.1–0.2	0.9–1.1	0.9–1.1	0.9–1.1
Negative	< 60	< 0.01	< 0.1	< 0.9	< 0.9	< 0.9

*IU* International units, *ml* Millilitre, *Max. protec.* Maximum. Data according to technical files provided by VIRCELL.

To optimize the DBS use for IgG measurements, the sensitivity, specificity and positive (PPV) and negative (NPV) predictive value of the tests were calculated for each of the six measurements in DBS of the 42 children and adolescent under study with paired plasma/DBS, considering as reference the results obtained in plasma samples. To obtain an optimal cut-off that offered the maximum sensitivity and/or specificity in DBS for the detection of protective IgG against each pathogen, ROC curves were generated with R software, as described in Supplementary Table 1. Indeterminate values were excluded from the final calculations. Statistical analyses were carried out with Stata.

### Ethical considerations

The project was approved by the Human Subjects Review Committees at Monkole Hospital/University of Kinshasa (DRC) and Hospital Ramón y Cajal (Madrid, Spain). Informed consent was obtained from parents or guardians of enrolled participants. Children and adolescents also provided assent after parental consent when they could understand the meaning of participation in the study. All methods were carried out in accordance with relevant guidelines and regulations. Patients’ names were codified at sampling to maintain confidentiality.

### Statistical analysis

GraphPad Prism v8.0.1. was used for all statistical analysis, considering statistically significant *p*-values of < 0.05, and using Chi-square or Fisher exact test as appropriate. We used Fisher exact test when the theoretical frequencies for categorical variables of HIV-infected and uninfected children in the contingency table had an absolute value < 5.

## Results

### Study population

The median age of the 143 patients was eleven years old, and 52.4% were male. Among them, 52 (36.4%) were HIV-infected, most (80.8%) presented viral load (VL) above 1000cp/ml, and only half (52.4%) were ART experienced since they were receiving antiretroviral treatment at sampling (Table 2).

**Table 2:** Characteristics of the study population from Kinshasa (DRC).

	With paired DBS/plasma (%)	Only with DBS (%)	Total
Total available samples	42 (100)	101 (100)	143 (100)
Male gender	19 (45.2)	56 (55.4)	75 (52.4)
Median age at sampling [IQR]	14 [12−17]	9 [3–12.8]	11 [5–14]
< 1	1 (2.4)	18 (17.8)	19 (13.3)
≥ 1–5	0	12 (11.9)	12 (8.4)
> 5–10	5 (11.9)	22 (21.8)	27 (18.9)
> 10–15	16 (38.1)	32 (31.7)	48 (33.5)
> 15	18 (42.9)	16 (15.8)	34 (23.8)
Unknown	2 (4.7)	1 (1)	3 (2.1)
**Confirmed HIV status**			
HIV+	38 (90.5)	14 (13.9)	52 (36.4)
HIV−	4 (9.5)	87 (86.1)	91 (63.6)
**ARV experience and VL**			
ARV experience	39 (92.9)	23 (22.8)	75 (52.4)
≥ 1000 cp/ml	31 (73.8)	11 (10.9)	42 (29.4)
**Mother’s HIV status**			
HIV+	17 (40.5)	43 (42.6)	60 (41.9)
HIV−	4 (9.5)	24 (23.7)	28 (19.6)
Unknown	21 (50)	34 (33.7)	55 (38.5)

Information extracted from clinical files. *DRC* The Democratic Republic of the Congo, *DBS* Dried blood samples, *IQR* Interquartile range, *ART* Antiretroviral treatment, *VL* *HIV-1* Viral load; Viral load quantified by Cobas®v2.0 (ROCHE), limit of quantification 20cp/ml. Age in years. cp/ml, HIV-1 RNA copies per millilitre of plasma corrected from the DBS cp/dot considering the patient’s haematocrit^22^.

### Immunization level in plasma of the paediatric population

**Figure 1:**
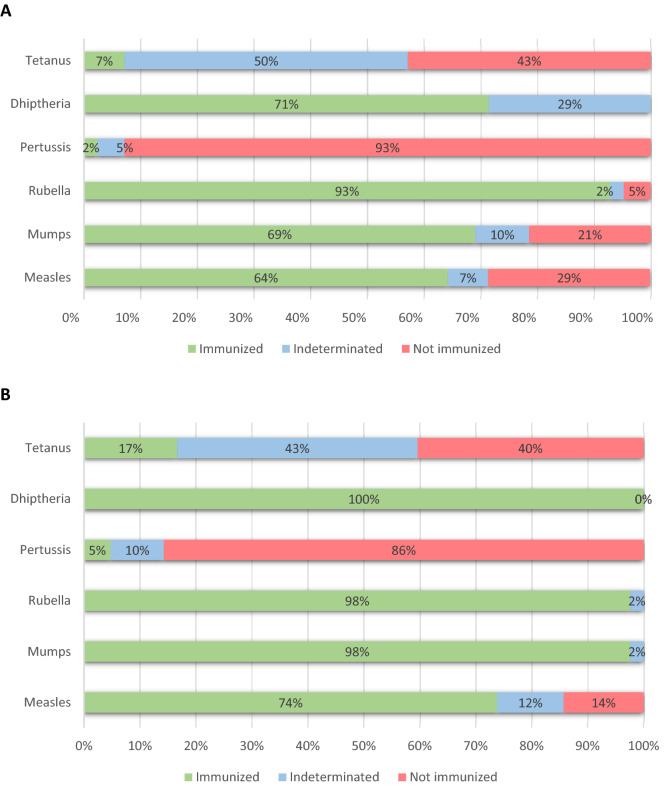
Pathogen immunity in 42 paediatric and adolescents from Kinshasa with available plasma (**A**) or DBS (**B**) considering the VIRCELL cut-off.

Protective IgG levels greatly varied for each pathogen in the study cohort. Different protection coverage rates, or % of subjects with immune response against an infection and/or disease^23^, was found in the 42 children and adolescents with available plasma for rubella (93%), diphtheria (71%), mumps (69%), measles (64%), tetanus (7%) and pertussis (2%) in plasma (Fig. 1A). Therefore, we found that 93% of children/adolescents did not show IgG protection against pertussis, 43% against tetanus, 29% against measles and 21% against mumps. The percentage of patients with indeterminate results in plasma was variable, ranging from 2% for rubella to 50% for tetanus (Fig. 1A). Indeterminate values were excluded from the final calculation.

### Cut-off calculation for each pathogen using DBS

To evaluate the validity of DBS to monitor the immune protection, we also analyzed the presence of protective IgG against the six pathogens in DBS from the 42 subjects with available paired DBS/plasma specimens (Fig. 1B). The sensitivity of each VirClia-IgG test for the detection of antibodies in DBS, considering the same cut-off for plasma (gold-standard sample) established by the manufacture’s, was 100% for pertussis, diphtheria and mumps, 97.4% for rubella and lower for measles (81.8%) and tetanus (66.7%). Specificity was very high for pertussis (97.6%), but decreased for tetanus (87.2%), measles (40%), mumps (7.7%), diphtheria and rubella (0% each). The PPV ranged from 28.6% (tetanus) to 92.7% (rubella) (Table 3).

**Table 3:** Results of VirClia-IgG test for the detection of protective IgG against six pathogens responsible for vaccine-preventable diseases using dried blood samples from 42 paediatric patients.

		MEASLES					MUMPS					RUBELLA				
		*Cut-off*	Sen	Spe	PPV	NPV	*Cut-off*	Sen	Spe	PPV	NPV	*Cut-off*	Sen	Spe	PPV	NPV
PLASMA	> 1.1	**100%**	**100%**	**100%**	**100%**	> 1.1	**100%**	**100%**	**100%**	**100%**	> 1.1	99%	**100%**	**100%**	**100%**	**100%**
DBS with PLASMA cut-off	> 1.1	81.8%	40%	71%	54.6%	> 1.1	**100%**	7.7%	70.7%	**100%**	> 1.1	97.4%	0%	92.7%	0%	0%
DBS	Max Sen	≥ 0.73	**100%**	13.3%	67.5%	**100%**	> 1.1	**100%**	7.7%	70.7%	**100%**	> 1.04	**100%**	0%	95%	0%
	Max Esp	≥ 2.83	55.6%	**100%**	**100%**	55.6%	≥ 6.18	37.9%	100%	**100%**	41.9%	≥ 3.97	43.6%	**100%**	**100%**	12%
	Optimal Sen and Spe	≥ 2.7	59.3%	93%	94%	56%	≥ 5.19	55.2%	76.9%	84%	43%	≥ 1.84	92.3%	66.7%	97.3%	40%

		DHIPTHERIA					PERTUSSIS					TETANUS				
		*Cut-off* (IU/ml)	Sen	Spe	PPV	NPV	*Cut-off* (IU/ml)	Sen	Spe	PPV	NPV	*Cut-off* (IU/ml)	Sen	Esp	PPV	NPV
PLASMA	> 0.01	**100%**	**100%**	**100%**	**100%**	> 120	**100%**	**100%**	**100%**	**100%**	> 0.2	96%	**100%**	**100%**	**100%**	**100%**
DBS with PLASMA cut-off	> 0.01	**100%**	0%	71.4%	0%	> 120	**100%**	97.6%	50%	**100%**	> 0.2	66.7%	87.2%	28.6%	97.1%	97.1%
DBS	Max Sen	≥ 0.03	**100%**	25%	76,9%	**100%**	≥ 586	**100%**	**100%**	**100%**	**100%**	≥ 0.11	**100%**	46.2%	12.5%	**100%**
	Max Spe	≥ 0.1	36.7%	**100%**	**100%**	38.7%	≥ 586	**100%**	**100%**	**100%**	**100%**	≥ 0.5	33.3%	**100%**	10%	95.1%
	Optimal Sen and Spe	≥ 0.09	46.7%	83.3%	78.5%	38.5%	≥ 586	**100%**	**100%**	**100%**	**100%**	≥ 0.24	66.7%	89.7%	33.3%	97.2%

*IU* International units, *ml* Millilitre, *PPV* Positive predictive value, *NPV* Negative predictive value, *DBS* Dried blood spots sample, *Max* Maximum, *Sen* Sensitivity, *Spe* Specificity. The cut-offs provided by VIRCELL for plasma have been taken as reference (see Table 1).

100% values are in bold.

A new cut-off (optimal cut-off) was calculated for each test to achieve optimal sensitivity and/or specificity in the detection of IgG using DBS. The application of optimal cut-off for each pathogen allowed to increase the PPV and NPV of the tests from the study cohort when using DBS (Table 3).

### Comparison of immunization results in DBS vs. plasma in the 42 children and adolescents with paired samples

The agreement in the immune protection rate for the 6 pathogens under study in the 42 children with paired DBS/plasma specimens varied according to the considered cut-off value showed in Table 3. Supplementary Table 2 provides the specific correlation between plasma and DBS in each child with paired DBS/plasma, highlighting these HIV positive children and considering different cut-offs: VIRCELL (see Table 1), optimum (see Table 3), and maximum specificity (see Table 3).

**Figure 2:**
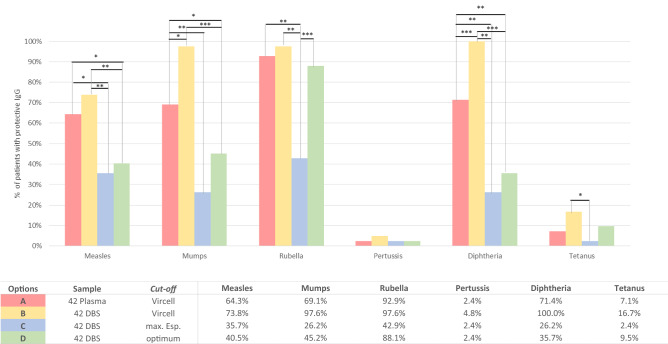
Pathogen immunity in 42 children and adolescents from Kinshasa with paired plasma/DBS considering different cut-offs. The plasma cut-off provided by VIRCELL for each pathogen is shown in Table 1. Options A and B, with the VIRCELL cut-off according to Table 1. Options C and D, with the maximum specificity or optimum cut-offs calculated for each pathogen, as indicated in Table 3. Statistically significant differences found: *p = 0.01–0.03; **p = 0.001–0.003; ***p < 0.0001.

When using the plasma cut-off for DBS, the percentage of immunized children in DBS for all pathogens was higher than when using plasma (Fig. 2 options A vs. B), with significant differences for 2 of them (+ 28.6% for mumps and diphtheria). By contrast, the observed protection was only + 2.4% for pertussis, + 4.8% for rubella and + 9.5% for tetanus and measles.

When we applied in DBS the cut-off providing maximum (100%) specificity (Fig. 2 option C), the rate of protected children for all pathogens was underestimated from 2–3 fold times vs. values provided in plasma with VirClia cut-off for 5 pathogens: rubella (-50%), diphtheria (− 45.2%), mumps (− 42.9%), measles (− 28.6%) and tetanus (− 4.8%). The only exception was pertussis, showing a similar low number of protected subjects than in plasma.

When the optimal cut-off showed in Table 3 for each pathogen was applied in DBS (Fig. 2 option D), the percentage of immunized among these 42 children was lower than using plasma to diphtheria (− 35.7%), mumps and measles (− 23.8%) and rubella (− 4.8%), was slightly higher (+ 2.4%) to tetanus, and similar to pertussis.

### Estimated immunization level in DBS of 143 children and adolescents

To study the protection coverage rates to 6 vaccine-preventable diseases in the whole study population from Kinshasa, we evaluated the percentage of children with protective IgG against each pathogen under study in the 143 DBS using cut-off providing maximum (100%) specificity (corresponding to 0% false positives) described at Table 3. We decided to use this cut-off to guarantee that all patients identified as immunized in the whole study cohort were protected, avoiding overestimation in the percentage of immunized patients. We did not use the plasma cut-off due to the previously observed overestimation of the percentage of immunized for all pathogens (Fig. 2 option B). Supplementary Table 3 provides the specific correlation between levels of reactive IgG in DBS from 143 children according to their HIV status and considering both maximum specificity and optimal cut-offs provided in Table 3.

**Figure 3:**
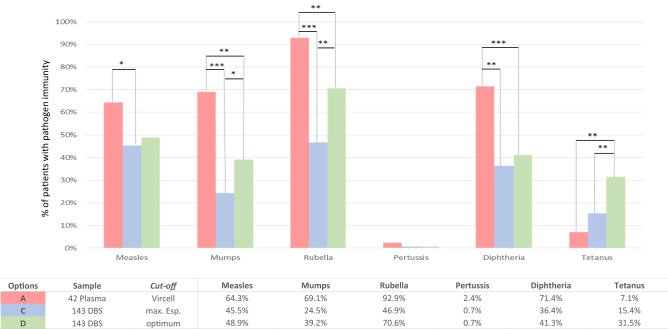
Comparison of the percentage of patients with pathogen immunity in the study population (42 plasmas vs. 143 DBS) using the optimal and maximum specificity cut-offs. The plasma cut-off provided by VIRCELL for each pathogen is shown in Table 1. The optimal or maximum specificity cut-offs calculated for each pathogen are indicated in Table 3. Statistically significant differences found: *p = 0.01–0.03; **p = 0.001–0.003; ***p < 0.0001.

With this 100% specificity cut-off, protective IgG were detected in less than half of the 143 studied patients: 46.9% for rubella, 45.5% for measles, 36.4% for diphtheria, 24.5% mumps, and 0.7% for pertussis. As it happened with the immunization values obtained when comparing the 42 paired samples DBS vs. plasma, immunization rates were between 2 and 3 times lower than those observed in plasma (gold standard) comparing data from the 143 DBS vs. 42 plasma (15.4% vs.7.1%), except for tetanus, where the percentage of immunized patients doubled (Fig. 3).

### Impact of HIV infection in protection rate to 6 vaccine-preventable pathogens

**Figure 4:**
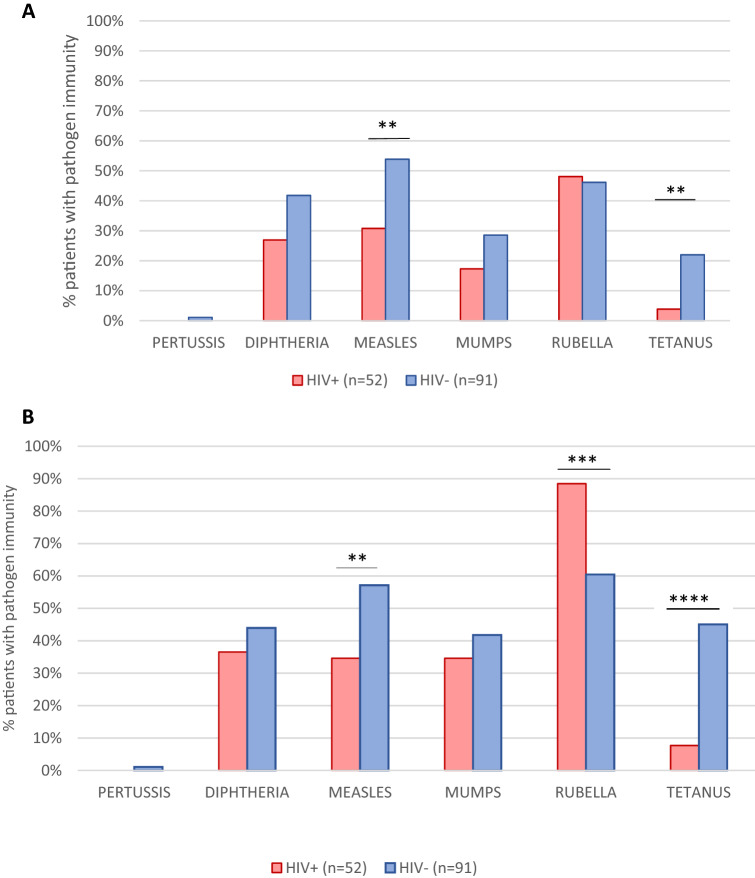
Percentage of patients with pathogen immunity according to the HIV status in 143 children and adolescents using DBS and cut-off of maximum specificity (**A**) and optimal (**B**). HIV+ , HIV-infected patients; HIV−, HIV-uninfected patients; % patients with protective IgG, percentage protection of patients against each of the 6 pathogens according to their reactive IgG levels. With asterisks, statistically significant differences found: **p = 0.00095, ***p = 0.0004, ****p < 0.0001.

Based on the analysis in the 143 DBS using the cut-off providing maximum (100%) specificity (Fig. 4A), we explored the impact of HIV infection in the protection coverage rates to the 6 vaccine-preventable diseases. Protective IgG to each pathogen were measured in 52 HIV-infected vs. 91 uninfected children of the study population from Kinshasa. We observed lower vaccine protection in HIV infected children except for rubella, which was slightly higher. The differences were statistically significant for measles (30.8% vs. 53.8%, *p* = 0.008) and for tetanus (3.8% vs. 22%, *p* = 0.0034). When the optimal cut-off was applied (Fig. 4B) the significant differences between HIV-infected and uninfected were maintained in measles (34.6% vs. 57.1%, *p* = 0.0095) and tetanus (7.7% vs. 45.1%, *p* < 0.0001). However, a significant difference was also observed for rubella (88.5% vs. 60.4%, *p* = 0.0004).

## Discussion

The evaluation of immunization programs is key to identify areas of suboptimal vaccination coverage^24^. Our pilot study provides pioneer data related to the seroprotection to six infectious diseases preventable by vaccination in a cohort of children and adolescents living in Kinshasa (DRC) using samples collected during 2016–2018. The quantification of IgG levels in DBS did not present any difficulty compared to plasma, and we detected IgG in the plasma fraction present in the dried blood drop collected on Whatman paper, as we previously did for other pathogens^20,21,25–27^.

Our results reveal that IgG levels greatly varied for each pathogen in the study population, not reaching the percentage necessary to achieve herd immunity, considering this as the minimum number of IgG reactive individuals necessary to avoid the pathogen spreading in the population. We also observed lower vaccine protection in HIV infected children vs. uninfected. We confirmed the usefulness of DBS in the absence of plasma/serum to study seroprevalence against vaccinable-preventive diseases. The DBS use for seroprevalence surveillance studies with this approach, and after optimizing the cut-off value for each pathogen, would allow for targeted vaccination campaigns, which could potentially provide services to the communities most in need and reduce vaccine campaign cost. We also provided the guideline for the adaptation of VIRCELL assays from serum to DBS, and the optimal pre-analytical treatment of DBS specimens for clinical vaccine or seroepidemiological studies.

Control of airborne viral infections with a very high reproduction number, like measles, mumps or rubella, requires high vaccine coverage^28,29^. Herd immunity, or the minimum number of individuals with pathogen immunity necessary to avoid the pathogen spreading in the population, has been previously defined^30–34^. Herd immunity threshold differs across pathogens: 91–94% for measles, 86–93% for mumps, 83–94% for rubella, 75–80% for diphtheria, and 90–94% for pertussis^32^. However, herd immunity thresholds and reproduction number can vary slightly across authors for some pathogens^32,33^. *Clostridium tetani* is not communicable between human hosts and there is no threshold proportion of immune persons below 100% that can ensure total absence of tetanus from a community^33^

Despite measles is endemic in the DRC, and 91–94% herd immunity is required to halt its transmission, coverage not reached in many African countries^35^. Among the 42 children and adolescents from Kinshasa with available plasma, 7 out of 10 were immunized against mumps and 9 out of 10 against rubella (Fig. 2), despite the absence of both vaccines in the DRC vaccination schedule^36^, revealing that protection was due to pathogen exposition and not by vaccination. We found that 6 out of 10 were immunized against measles, whose vaccine is administered at 9 months of age in the RDC.

Antibodies against measles, mumps and rubella can be present for up to 20 years after trivalent vaccine administration^37^, decreasing in some children when the vaccines are given separately^38^. Although MMR vaccine-induced protection appears to persist at least into early adulthood, constant monitoring of protection against these pathogens is necessary^37^. In addition, it must be taken into account that the current vaccination schedule during the first year of life of older patients under study could differ from the current one given to younger participants, modifying the protection against some of the vaccine pathogens. Our data revealed similar immunization rates to measles (64%) in the study paediatric population from Kinshasa during 2016–2018 than in other paediatric cohorts in the DRC during 2013–2014^39^, being slightly higher than the vaccination coverage after the first and single dose of measles vaccine administered at 9 months of age reported by the WHO in that country (57%)^9^.

DTP3 coverage in the DRC, used as an indicator of the capacity of a country to provide immunization services, reached 57% in 2019^9^. However, we found different immunization rates for each pathogen in the 42 plasma from the study population, being 71.4% of them immunized against diphtheria, but only 7.1% against tetanus and 2.4% against pertussis. If patients protected against diphtheria had suffered from the disease was not reported in the available clinical files. The high vulnerability to tetanus and pertussis of the Congolese paediatric and adolescent population is consistent with the latest official data from the country^9^.

The interference with other infectious diseases affects the loss of seroprotection^40–44^. In this regard, we wanted to analyse the impact of HIV infection on seroprotection levels against the 6 pathogens in our paediatric cohort from Kinshasa. To avoid the loss of statistical robustness of the results due to the low sample size using plasma samples (only in 42 patients), we used DBS, the available sample in all 143 subjects under study. We considered the cut-off providing 100% specificity to guarantee that all patients identified as immunized were protected and to avoid overestimation in the rate of immunized children and adolescents in the complete study cohort. However, it is important to note that the cut-off providing 100% specificity in the detection of protective IgG for each pathogen in DBS might be always calculated in each study population with paired plasma/DBS samples and only be extrapolated to other population with similar prevalence for each analyzed infection.

The effect of HIV-1 exposure and antiretroviral treatment strategies in HIV-infected children on the immunogenicity of vaccines during infancy has been also reviewed, showing differences in protection^45^. Since HIV infection impairs humoral and cell-mediated immune functions early in infancy^46,47^, the poor antibody levels to different vaccines found in our study could be probably related to major immunological dysfunction involving both cellular and humoral responses in the children under study. Some authors have analyzed the persistence of antibodies to the vaccines in HIV-infected and HIV-exposed uninfected children who previously received these vaccines in routine clinical practice, also reporting lower antibody concentrations after vaccination against some vaccine-preventable diseases in HIV-infected vs. uninfected children^48^. Furthermore, low measles antibody concentration in HIV-infected children has been associated with severe immunodeficiency (< 25% CD4 + T cells, HIV VL ≥ 10,000 copies) in HIV-infected children, as well as to the short duration of antiretroviral treatment^48^. Unfortunately, CD4 data at sampling was not reported in the clinical files of the HIV-infected children under study.

Since children living with HIV present a higher risk of vaccine-preventable infectious diseases than non-infected^10^, vaccination with an adapted schedule, revaccination and periodic monitoring of their seroprotective status are recommended^13,48,49^. The low levels of seroprotection for all pathogens except rubella observed in the 52 HIV-infected patients vs. 91 HIV-uninfected could be explained by the lower capacity of the children with HIV to maintain the titers at adequate levels over time^48^.

Regarding measles, low persistence of antibodies to the measles vaccine has been observed mainly in HIV-infected but also in HIV-exposed children born to HIV-infected mothers^48,50^. The level of measles immunity at birth in both HIV-infected and HIV-1 exposed children would depend on the level of antibodies in the HIV-infected mother and on the extent of placental transfer^50^. In HIV-infected and exposed children, maternal HIV infection might reduce levels of measles antibodies in newborns, and the low levels of measles antibodies at birth would render children susceptible to measles infection at an early age^50^. In HIV-infected children increases the number of CD4 + T cells and B cells during ART, whereas the total immunoglobulin levels decline. Despite immune reconstitution, antibodies against live-attenuated vaccines and wild-type natural virus strains disappear over time in up to 40% of children with HIV-1 infection^51^. Fewer HIV-infected children were protected after vaccination at 12 months than HIV-exposed but uninfected children have been reported, as well as an increase in the proportion of seropositive children increased with increasing age at vaccination among HIV-exposed but uninfected and HIV-unexposed children^52^. Another study showed that the majority of HIV-infected children vaccinated against measles in Morocco develop a suboptimal seroprotective titer^53^.

This study has several limitations. Firstly, plasma was only available in 42 of 143 subjects under study since we used retrospective and remaining samples from other studies in paediatric population in Kinshasa^21,54–56^. Unfortunately, the number of received doses in each enrolled child was not reported in the clinical files, preventing distinguishing the children and adolescents with protective IgG due to vaccination or due to infection. Secondly, the number of positive/negative samples for each pathogen in our study population was very uneven among the 42 with available plasma specimens, ranging from only 1 subject infected with pertussis to 39 infected to rubella. Thus, future similar studies should include a higher number of subjects and a similar number of infected and uninfected children per infection, for a better adjustment of the cut-off values for each pathogen. Thirdly, the cut-off values providing 100% specificity in DBS for each pathogen could not be appropriate for other cohorts with different prevalence of the studied vaccinable-preventive diseases. Cut-off values providing 100% specificity should be always calculated in paired DBS/plasma specimens for the same setting or study cohort with a similar prevalence of vaccinable-preventive diseases under study. In addition, the provided new cut-offs for DBS would be VIRCELL-specific and should not be extrapolated to other similar assays for seroprevalence studies.

## Conclusions

Our data reinforce the necessity to increase/improve vaccination coverage in Kinshasa, paying special attention to the HIV-infected paediatric population, with less capacity to maintain antibody titers at adequate levels. To prevent future outbreaks and to avoid preventable deaths from vaccination, we emphasize the urgency for periodic monitoring immunity to vaccine-preventable diseases globally and mainly in countries with limited resources. The use of DBS could help to expand antibody detection for monitoring seroprotection level coverage among children in resource-limited settings when plasma/serum collection is complex or absent. However, the cut-off values should be optimized when DBS are used for seroprevalence studies. Finally, we recommend carrying out new similar studies with a larger sample size with available paired plasma/DBS specimens per subject and vaccination data, and an equitable number of vaccinated and unvaccinated patients to assign a definitive cut-off value for each pathogen in DBS. The procedures described here could be incorporated into existing data collection protocols in the DRC to monitor vaccinable-preventive disease exposures, herd immunity, and effectiveness of immunization programs.

## Supplementary Information

Supplementary Information 1.

Supplementary Information 2.

Supplementary Information 3.
